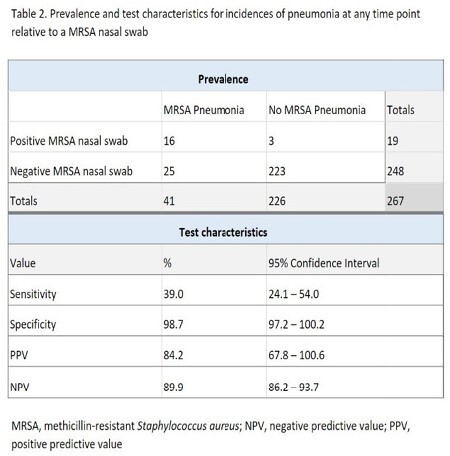# 513 Predictive Values of MRSA Nasal Swabs for Pneumonia in Burn ICU Patients

**DOI:** 10.1093/jbcr/irae036.148

**Published:** 2024-04-17

**Authors:** Zachary Carlson, Robyn N Stoianovici, Sierra R Young, Erin L Louie

**Affiliations:** UC Davis Medical Center, Sacramento, CA; UC Davis Health, Sacramento, CA; UC Davis Medical Center, Sacramento, CA; UC Davis Health, Sacramento, CA; UC Davis Medical Center, Sacramento, CA; UC Davis Health, Sacramento, CA; UC Davis Medical Center, Sacramento, CA; UC Davis Health, Sacramento, CA

## Abstract

**Introduction:**

Sepsis in burn-injured patients remains a leading cause of mortality in those who survive initial resuscitation. Methicillin-resistant Staphylococcus aureus (MRSA) is a common causative pathogen for pneumonia in burn-injured patients. Studies evaluating the predictive value of MRSA nasal swabs for pneumonia have largely excluded the burn-injured population.

**Methods:**

Patients 18 years or older admitted to the Burn ICU at a tertiary medical center from 2016 to 2021 were included if they had any burns, a pneumonia ICD-10 code, an MRSA nasal swab obtained during admission, and any respiratory cultures associated with at least five consecutive days of antibiotics.

**Results:**

There were 267 instances of pneumonia across 136 patients included. MRSA nasal swabs had an overall sensitivity of 39%, specificity of 98.7%, positive predictive value (PPV) of 84.2%, and negative predictive value (NPV) of 89.9%. MRSA nasal swabs obtained less than seven days from antibiotic initiation had a specificity of 98.6% and NPV of 98.6%; meanwhile, swabs obtained at least seven days from antibiotic initiation had a specificity of 98.7% and NPV of 86.4%.

**Conclusions:**

The high specificity and NPV indicate that negative MRSA nasal swabs obtained less than seven days from antibiotic initiation may be used to de-escalate anti-MRSA antibiotics in clinically stable burn-injured patients with suspicion of pneumonia. The decrease in NPV suggests that it may be beneficial to obtain a repeat swab periodically or upon suspicion of pneumonia.

**Applicability of Research to Practice:**

This study is the first to measure the sensitivity, specificity, PPV, and NPV of the MRSA PCR nasal swab in a cohort comprised only of patients admitted to a burn intensive care unit. The findings support the use of the MRSA nasal swab as a tool to aid in the decision to initiate or de-escalate anti-MRSA antimicrobials for pneumonia.